# Early-Onset Amiodarone Pulmonary Toxicity at a Standard Maintenance Dose Masquerading as Decompensated Heart Failure: A Case Report and Literature Review

**DOI:** 10.7759/cureus.109617

**Published:** 2026-05-25

**Authors:** M. Nour Chabalout, Afnan Chaudhry, Ibrahim Inanc, Saad Khan, Mohammad Alazzeh

**Affiliations:** 1 Internal Medicine, Tower Health Medical Group, Phoenixville, USA; 2 Internal Medicine, Phoenixville Hospital, Phoenixville, USA; 3 Internal Medicine, Tower Health Medical Group, Philadelphia, USA

**Keywords:** amiodarone pulmonary toxicity, drug-induced lung disease, heart failure with preserved ejection fraction, interstitial lung disease, low-dose amiodarone

## Abstract

Amiodarone is a widely used antiarrhythmic agent. Pulmonary toxicity is its most feared non-cardiac complication and is classically associated with high doses and prolonged duration of therapy. We report a case of early-onset amiodarone pulmonary toxicity (APT) developing after approximately one month of standard-dose amiodarone therapy (400 mg/day) in the setting of concurrent decompensated heart failure with preserved ejection fraction (HFpEF), which substantially complicated the diagnostic evaluation.

A 71-year-old male with paroxysmal atrial fibrillation, nonischemic cardiomyopathy, and multiple comorbidities was initiated on amiodarone 400 mg/day in April. Within one month, he developed progressive dyspnea on exertion and productive cough. He presented to the emergency department in September with acute hypoxic respiratory failure (SpO₂ 86% on room air), weight gain, and bilateral lower extremity edema approximately five months after symptom onset. CT of the chest demonstrated bilateral ground-glass and consolidative opacities with upper lobe predominance and interlobular septal thickening. The initial working diagnosis of decompensated HFpEF was supported by elevated BNP and peripheral edema; intravenous furosemide led to the resolution of edema but no improvement in oxygenation. APT was subsequently suspected, and intravenous methylprednisolone was initiated, resulting in marked clinical improvement. The patient was discharged on room air on a prolonged prednisone taper. At the three-month follow-up, he demonstrated complete symptomatic resolution, marked radiological improvement, and normalization of BNP.

This case adds to the existing evidence that clinically significant APT may occur at standard maintenance doses and within a shorter timeframe than traditionally recognized. Coexistent HFpEF poses a major diagnostic challenge given the substantial overlap in clinical and radiological features. The possibility of concurrent rather than competing pathology should always be considered. Clinicians should maintain a high index of suspicion for APT in any patient on amiodarone with progressive respiratory symptoms, irrespective of dose or duration of exposure.

## Introduction

Amiodarone is a class III antiarrhythmic drug that remains indispensable in the management of both ventricular and supraventricular arrhythmias, including atrial fibrillation (AFib) and flutter. Its efficacy, however, is offset by a well-recognized spectrum of adverse effects involving multiple organ systems. Among these, amiodarone pulmonary toxicity (APT) is particularly consequential, carrying significant morbidity and a mortality rate ranging from approximately 9% in cases of chronic pneumonitis to as high as 50% in those who develop acute respiratory distress syndrome [1].

The reported incidence of APT varies widely across studies. The 2023 ACC/AHA/ACCP/HRS Atrial Fibrillation Guidelines cite an incidence of 1-2% for clinically significant pulmonary toxicity [1]. However, a review published in JAMA reported a range of 1-17%, with the wide variation attributed to differences in dosing regimens and patient populations [2]. A large population-based Canadian study of 6,460 amiodarone users reported an overall incidence of 3.87%, with age-standardized rates of 28.3 per 1,000 person-years in men and 16.0 per 1,000 person-years in women [3]. More recently, a nationwide Israeli study of patients with AFib on contemporary low-dose amiodarone identified interstitial lung disease (ILD) in 2.0% over a mean follow-up of 4.2 years [4].

APT encompasses several clinicopathological patterns, with chronic interstitial pneumonitis being the most common. The onset is typically insidious, manifesting with progressive dyspnea, nonproductive or productive cough, and constitutional symptoms. The condition can also present as an acute hypersensitivity pneumonitis or, rarely, as organizing pneumonia or diffuse alveolar damage [5]. Established risk factors include older age, higher daily maintenance doses (particularly >400 mg/day), greater cumulative dose, pre-existing pulmonary disease, male sex, renal impairment, and prior cardiothoracic surgery [2,3].

Timing of onset is notably variable. While toxicity can develop within days of initiation or years into therapy, the highest incidence has been reported within the first 12 months, with one landmark case series of 573 patients demonstrating that 55% of cases were diagnosed within the first year [6]. Cases arising from standard maintenance doses (≤400 mg/day) within a short period of initiation are infrequently described in the contemporary literature, though emerging data suggest that the risk may not be as negligible as previously assumed.

The diagnostic challenge of APT is further amplified when it coexists with decompensated heart failure. Both conditions share overlapping clinical features, including dyspnea, hypoxemia, bilateral pulmonary infiltrates, and elevated natriuretic peptides. We present a case that illustrates this diagnostic dilemma and highlights the importance of maintaining a broad differential - and considering concurrent pathology - in patients on amiodarone with progressive respiratory decline.

## Case presentation

History and presentation

A 71-year-old male with a past medical history of paroxysmal AFib, nonischemic cardiomyopathy (NICM), type 2 diabetes mellitus (T2DM), benign prostatic hyperplasia, and hypertension presented to the emergency department in September with acute hypoxic respiratory failure. He reported a several-month history of progressive shortness of breath on exertion, dizziness, and productive cough, with symptoms dating back to May. The patient reported a history of a prior left lower extremity deep vein thrombosis and was maintained on long-term anticoagulation with rivaroxaban 20 mg once daily. He was a social drinker and an occasional cigar smoker (once to twice per week). He had no prior chest imaging and was not on home oxygen.

Amiodarone 400 mg daily had been initiated in April for the management of AFib and flutter in the setting of underlying NICM. Respiratory symptoms began approximately one month after drug commencement (May) and gradually worsened over the following months. For the one to two weeks preceding admission, the patient noted significant symptom acceleration and modest weight gain in addition to bilateral leg swelling. He denied chest pain, palpitations, paroxysmal nocturnal dyspnea (PND), fever, or chills. He reported sleeping on two pillows and needing to lie on his side for comfort. It is noteworthy that the patient had not been initiated on any guideline-directed medical therapy (GDMT) for heart failure prior to this admission, including no diuretic, angiotensin-converting enzyme (ACE) inhibitor, angiotensin receptor blocker (ARB), or sodium-glucose cotransporter 2 (SGLT2) inhibitor. His home medications included metoprolol succinate 50 mg once daily, rivaroxaban 20 mg once daily, and rosuvastatin 10 mg once daily. Amiodarone had been discontinued approximately one week prior to admission following successful catheter ablation of his AFib in early September.

On arrival to the emergency department, the patient was found to be hypoxic with an oxygen saturation of 86% on room air, requiring supplemental oxygen at 4 L/min via nasal cannula to achieve SpO₂ 93%.

Physical examination

The patient was alert and oriented but in moderate respiratory distress. Vital signs revealed a blood pressure of 135/80 mmHg, respiratory rate of 24 breaths per minute, temperature of 36.9°C, and SpO₂ 93% on 4 L/min nasal cannula. There was no pallor, jaundice, or elevated jugular venous pressure. Chest auscultation revealed bilateral fine crackles scattered throughout the lung fields without wheezing or rhonchi. Heart sounds were regular with normal S1 and S2, and no murmurs or added sounds. The abdomen was soft, nontender, and nondistended. There was mild bilateral lower extremity pitting edema (1+) without clinical signs of deep vein thrombosis. Peripheral pulses were equal and symmetric.

Investigations

Electrocardiogram showed normal sinus rhythm with no ST-T abnormalities or ischemic signs. Laboratory evaluation is summarized in Table 1.

**Table 1:** Laboratory results on admission. Troponin values are reported in ng/L (high-sensitivity assay). Reference ranges reflect standard institutional laboratory values. BNP, B-type natriuretic peptide; CRP, C-reactive protein; WBC, white blood cell

Parameter	Value	Reference Range
WBC count	10.0 × 10⁹/L	4.0–11.0 × 10⁹/L
Hemoglobin	15 g/dL	13.5–17.5 g/dL
Platelets	262 × 10⁹/L	150–400 × 10⁹/L
Sodium	138 mEq/L	135–145 mEq/L
Potassium	4.1 mEq/L	3.5–5.0 mEq/L
Magnesium	2.1 mg/dL	1.7–2.2 mEq/L
Troponin (initial)	142 ng/L	<52 ng/L
Troponin (3-hour)	120 ng/L	<52 ng/L
BNP	485 pg/mL	<100 pg/mL
CRP	0.7 mg/L	<10 mg/L
Procalcitonin	0.1 ng/mL	<0.25 ng/mL
Sputum culture	Negative	—

The troponin was mildly elevated on admission (142 ng/L) with a non-rising pattern at three hours (120 ng/L). In the absence of chest pain or electrocardiographic evidence of ischemia, this was attributed to demand ischemia in the setting of acute hypoxic respiratory failure rather than an acute coronary syndrome.

The chest radiograph demonstrated bilateral interstitial infiltrates (Figure 1). A CT of the chest with pulmonary angiography excluded pulmonary embolism but revealed widespread bilateral ground-glass and consolidative opacities with upper lobe predominance that appeared relatively symmetric, with associated interlobular septal thickening (Figure 2). Pulmonary function testing and bronchoscopy with bronchoalveolar lavage (BAL) were not performed during this admission.

**Figure 1:**
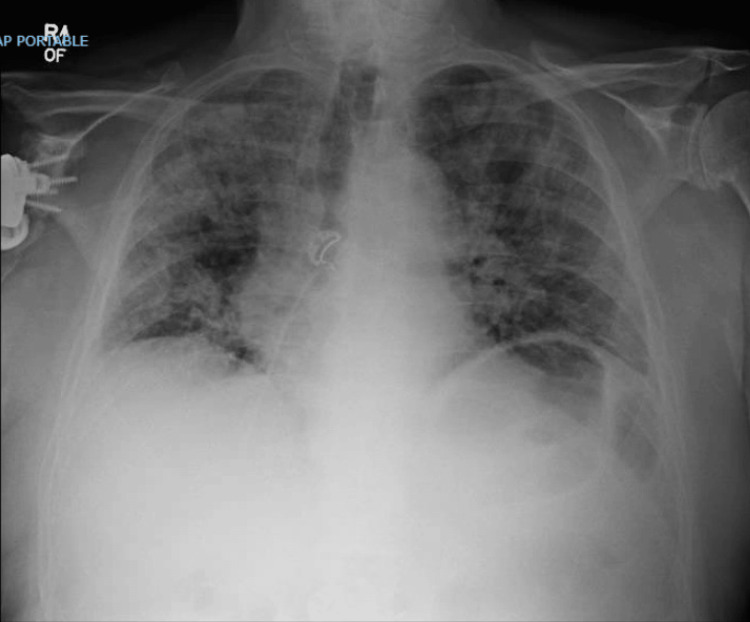
Anteroposterior chest radiograph on admission demonstrating bilateral interstitial infiltrates.

**Figure 2:**
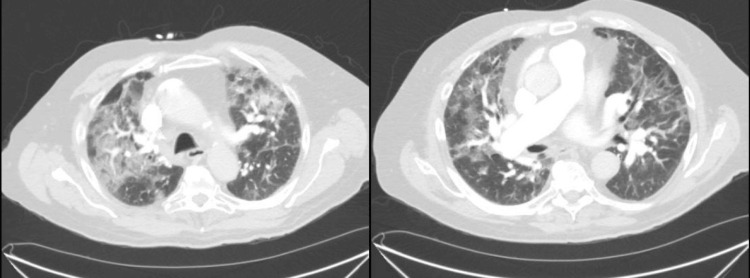
CT of the chest on admission showing widespread bilateral ground-glass opacities and consolidation with upper lobe predominance, relative symmetry, and interlobular septal thickening.

Testing for infectious causes including *Legionella*, *Streptococcus pneumonia*, and viral respiratory panel was negative.

A transthoracic echocardiogram was technically limited due to poor acoustic windows but demonstrated preserved left ventricular systolic function with an ejection fraction of 55-60%. There was mitral annular and valvular calcification with mild mitral regurgitation, aortic valve sclerosis with mild aortic regurgitation but no stenosis, and mild tricuspid regurgitation. These findings were unchanged compared to a prior study conducted two years ago.

Clinical course

The patient was initially admitted with a working diagnosis of acute hypoxic respiratory failure in the context of decompensated heart failure with preserved ejection fraction (HFpEF), supported by his symptom profile, elevated B-type natriuretic peptide ([BNP] 485 pg/mL), bilateral infiltrates on imaging, and lower extremity edema. Intravenous furosemide 40 mg twice daily was initiated alongside supplemental oxygen. Home medications, including metoprolol succinate 50 mg daily and rivaroxaban 20 mg daily, were continued.

After two days of diuretic therapy, bilateral lower extremity edema resolved; however, the patient’s respiratory status and oxygen requirements remained unchanged. This clinical dissociation prompted reconsideration of the diagnosis. The constellation of findings - temporal relationship between amiodarone initiation (April) and symptom onset (May), upper-lobe predominant CT pattern atypical for cardiogenic edema, failure to respond to diuresis, suppressed inflammatory markers (CRP 0.7 mg/L; procalcitonin 0.1 ng/mL), and negative sputum culture - raised a strong suspicion for APT. Importantly, while the response to diuresis supported a concurrent element of volume overload from decompensated HFpEF, the persistent hypoxemia suggested that APT was the predominant driver of respiratory compromise.

Intravenous methylprednisolone was initiated at 40 mg every eight hours, subsequently reduced to every 12 hours. Marked clinical improvement was observed within 48 hours of steroid initiation, with progressive decline in oxygen requirements. The patient was discharged on room air with SpO₂ 95%, with a prednisone taper initiated at 60 mg daily, reduced by 10 mg every five days over a total duration of one month. GDMT for heart failure was optimized at discharge with the addition of spironolactone and empagliflozin.

At the three-month follow-up with pulmonology, the patient reported complete resolution of dyspnea. Repeat CT of the chest demonstrated marked improvement in bilateral infiltrates (Figure 3). Repeat BNP was 81 pg/mL. The patient remained in sinus rhythm following his catheter ablation.

**Figure 3:**
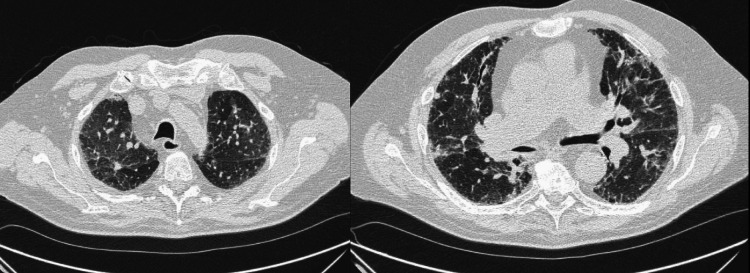
Follow-up CT of the chest at three months demonstrating marked improvement in the previously noted bilateral ground-glass opacities and consolidation, consistent with resolution of amiodarone pulmonary toxicity following steroid therapy and drug withdrawal.

## Discussion

This case presents several noteworthy clinical observations. First, it documents clinically significant APT developing after approximately one month of amiodarone at a standard maintenance dose of 400 mg/day. Second, the concurrent presence of decompensated HFpEF created a compelling diagnostic overlap that substantially delayed recognition of the drug-induced pulmonary process. Third, both conditions likely coexisted in this patient, with decompensated HFpEF contributing to the volume-overloaded state and APT being the predominant driver of persistent hypoxemia. Fourth, the patient demonstrated a favorable clinical response to corticosteroid therapy with complete resolution at three months.

The conventional understanding of APT frames it primarily as a complication of higher doses (>400 mg/day) and prolonged treatment durations. Established risk factors include daily maintenance doses exceeding 400 mg, greater cumulative dose, older age, male sex, and pre-existing pulmonary disease [2,3]. One landmark retrospective analysis found no cases of APT in patients receiving a mean daily dose below 305 mg, lending support to the notion that a minimum dose threshold may exist [6]. These observations have contributed to a clinical perception that lower maintenance doses carry a substantially reduced pulmonary risk.

The present case adds to an emerging body of evidence suggesting that this risk is not negligible even at standard or lower maintenance doses and may manifest earlier than previously appreciated. A contemporary nationwide Israeli study confirmed a trend toward increased ILD risk in AFib patients on contemporary low-dose amiodarone over a mean 4.2-year follow-up [4]. More strikingly, a recent substudy of the VANISH2 trial reported pulmonary disease in more than 15% of patients receiving 200 mg daily amiodarone for ventricular tachycardia, substantially exceeding historical estimates of 2-3% [7]. A summary of key studies examining APT incidence at lower or standard maintenance doses is presented in Table 2.

**Table 2:** Summary of selected studies examining APT incidence at lower or standard maintenance doses of amiodarone. AF, atrial fibrillation; APT, amiodarone pulmonary toxicity; ILD, interstitial lung disease; RCT, randomized controlled trial; VT, ventricular tachycardia

Study	Year	Design	Population	N	Dose	Key Finding	Follow-Up
Vorperian et al. [8]	1997	Meta-analysis (4 RCTs)	Mixed (post-MI, HF, AF)	1,465	≤400 mg/day (mean 152–330)	Trend toward increased pulmonary toxicity vs placebo; not statistically significant	≥12 months
Sunderji et al. [9]	2000	Pooled analysis of placebo-controlled trials	Mixed cardiac	3,439	≤400 mg/day	Combined incidence of APT: 1.6%	Variable
Ott et al. [10]	2003	Retrospective case series	Elderly cardiac patients (mean age 77 years)	8 cases	200 mg/day	All 8 patients developed confirmed APT at 200 mg/day; 1 death from respiratory failure	Mean ~2 years on drug
Jackevicius et al. [3]	2011	Population-based retrospective cohort (Canada)	AF patients ≥65 years	6,460 users	≤200 and >200 mg/day	Overall incidence 3.87%; both dose subgroups showed elevated risk vs nonusers	1999–2007
Ruzieh et al. [11]	2019	Meta-analysis (43 RCTs)	Mixed cardiac	11,395	Low-dose subgroup analyzed	Pulmonary adverse events higher with amiodarone overall; low-dose subgroup did not reach statistical significance	Variable
Tsaban et al. [4]	2024	Nationwide matched cohort (Israel)	AF patients, median age 73.3 years	6,039 matched pairs	Low-dose (contemporary practice)	ILD in 2.0%; maximum absolute risk increase 1.8%; trend toward elevated risk	Mean 4.2 years
Nery et al. (VANISH2) [7]	2026	RCT substudy	VT with ischemic heart disease	Substudy cohort	200 mg/day	Pulmonary disease in >15%, substantially exceeding prior historical estimates of 2–3%	Not specified

With respect to timing, APT has been documented as early as the first days of treatment [5,6]. In the present case, symptom onset occurred within one month of drug initiation (May), though hospital presentation did not occur until September - approximately five months later - likely reflecting a gradual worsening exacerbated by concurrent cardiac decompensation. This timeline underscores the importance of distinguishing between symptom onset and time to formal diagnosis, which may differ substantially when a competing diagnosis is present.

The proposed pathophysiology of APT involves two principal mechanisms: a direct dose-dependent toxic effect mediated by oxidative injury to the alveolar epithelium and lysosomal phospholipid accumulation, and an indirect immune-mediated or hypersensitivity reaction that is thought to be dose- and duration-independent [5]. While the former mechanism is more consistent with higher cumulative exposures, the latter may account for cases such as ours, where toxicity manifests after a relatively brief exposure. However, in the absence of bronchoscopy, BAL, or histopathological confirmation, the precise mechanism in this case remains speculative. The upper-lobe predominant CT pattern, suppressed inflammatory markers, negative microbiological workup, and corticosteroid responsiveness are all consistent with an immune-mediated process, though they cannot definitively exclude other etiologies.

An important concept illustrated by this case is that APT and decompensated HFpEF need not be mutually exclusive diagnoses and indeed likely coexisted in this patient. The patient’s bilateral edema, weight gain, elevated BNP, and known NICM without prior GDMT all support genuine cardiac decompensation as a concurrent pathological process. The partial response to diuresis (resolution of edema) is consistent with this interpretation. At the same time, the persistent hypoxemia despite adequate diuresis, the upper-lobe CT predominance, and the dramatic corticosteroid response point to APT as the dominant contributor to respiratory failure.

It is also noteworthy that the patient had not been initiated on any GDMT for heart failure prior to admission, including no diuretic, ACE inhibitor, ARB, or SGLT2 inhibitor. This gap in therapy may have contributed to a chronically elevated filling pressure and volume-overloaded state, thereby lowering the threshold for acute decompensation and further obscuring the primary pulmonary diagnosis.

The elevated BNP of 485 pg/mL, while consistent with decompensated HFpEF, should be interpreted with caution as a sole discriminator. BNP elevation in APT is not uncommon and may reflect right ventricular pressure overload secondary to hypoxemia, pulmonary hypertension, or concurrent cardiac dysfunction, rather than exclusively reflecting left-sided filling pressures.

Additional features that ultimately distinguished APT as the primary driver included the CT distribution (bilateral upper-lobe predominance and ground-glass morphology, atypical for cardiogenic pulmonary edema, which is typically perihilar, basal, and lower-lobe predominant), preserved left ventricular ejection fraction, suppressed inflammatory markers, and the direct temporal relationship between amiodarone initiation and symptom onset.

This case underscores that in patients on amiodarone presenting with combined respiratory and volume-overload features, the clinician should consider the possibility of overlapping pathology rather than defaulting to a single unifying diagnosis. The failure of oxygenation to respond to adequate diuresis should serve as a pivotal clinical signal to broaden the differential.

The cornerstone of APT management is discontinuation of amiodarone, which had already been achieved in this patient one week prior to admission following successful AFib ablation. Despite drug cessation, corticosteroid therapy was deemed necessary given the severity of respiratory compromise. Intravenous methylprednisolone followed by a prolonged oral prednisone taper (initiated at 60 mg daily with a step-down reduction over one month) resulted in rapid and sustained clinical improvement. Although the evidence base for corticosteroids in APT remains largely observational, their use is widely supported and is generally recommended in moderate-to-severe cases [5]. The optimization of GDMT with the addition of an SGLT2 inhibitor and mineralocorticoid receptor antagonist at discharge addresses the previously undertreated cardiac substrate and may reduce the risk of future HFpEF decompensation.

The complete clinical, symptomatic, and radiological resolution at the three-month follow-up, with near-normalization of BNP (81 pg/mL), provides strong retrospective support for the diagnosis of APT as the predominant driver of the acute presentation. These outcomes are consistent with favorable prognoses reported in cases where APT is recognized and treated in a timely manner.

Strengths, limitations, and future directions

This case report has several strengths. The clinical course is well-documented with serial laboratory data, cross-sectional imaging at admission and three-month follow-up, and echocardiographic assessment, providing a comprehensive picture of disease evolution and treatment response. The inclusion of a focused literature review contextualizes the case within the broader evidence base on APT at standard maintenance doses. However, several limitations must be acknowledged. The diagnosis of APT remains presumptive, as bronchoscopy, BAL, pulmonary function testing including diffusion capacity (DLCO), and histopathological confirmation were not obtained. Baseline pulmonary imaging and spirometry prior to amiodarone initiation were unavailable, precluding formal exclusion of pre-existing lung disease. As a single case report, generalizability is inherently limited, and causal inference must be interpreted with caution. Future prospective studies are needed to better characterize the incidence, risk factors, and time-to-onset of APT at standard maintenance doses, including 400 mg/day, particularly in patients with underlying structural heart disease and heart failure. Development and validation of a standardized clinical scoring tool incorporating imaging characteristics, temporal drug exposure, and biomarkers may facilitate earlier and more accurate diagnosis of APT in patients with concurrent cardiopulmonary pathology, where the diagnostic overlap is greatest.

## Conclusions

This case demonstrates that clinically significant APT may develop at a standard amiodarone maintenance dose of 400 mg/day and within a short period of drug initiation. While the diagnosis remains presumptive in the absence of bronchoscopy, BAL, or pulmonary function testing, the clinical course, including characteristic imaging findings, failure to respond to diuresis alone, and marked corticosteroid responsiveness, is strongly consistent with APT. Importantly, concurrent decompensated HFpEF likely contributed to the clinical presentation and should be viewed as a co-existing rather than a competing diagnosis. The absence of GDMT prior to admission may have facilitated volume overload and delayed clinical recognition. Clinicians should maintain a high index of suspicion for APT regardless of dose or treatment duration and should consider the possibility of dual pathology in patients with underlying heart failure who present with progressive respiratory symptoms while on amiodarone. When APT is suspected, amiodarone discontinuation remains the mainstay of treatment, and corticosteroids are generally preserved for severe cases.
